# Effects of Whole-Body Stretching Exercise during Lunch Break for Reducing Musculoskeletal Pain and Physical Exertion among Healthcare Professionals

**DOI:** 10.3390/medicina59050910

**Published:** 2023-05-10

**Authors:** Raee Saeed Alqhtani, Hashim Ahmed, Adel Alshahrani, Abdur Raheem Khan, Ashfaque Khan

**Affiliations:** 1 Department of Medical Rehabilitation Sciences-Physiotherapy Program, College of Applied Medical Sciences, Najran University, Najran 55461, Saudi Arabia; 2 Department of Physiotherapy, Integral University, Lucknow 226026, India

**Keywords:** pain, physical exertion, stretching, lunch break, whole-body, musculoskeletal discomfort

## Abstract

*Background and Objectives*: To investigate the effect of whole-body stretching (WBS) exercise during lunch break for reducing musculoskeletal pain and physical exertion among healthcare professionals. *Methods*: Full-time healthcare professionals working in hospitals with more than one year of experience were invited to participate. Sixty healthcare professionals (age 37.15  ±  3.9 Years, height 1.61  ±  0.04 m, body mass 67.8  ±  6.3 kg, and BMI 26.5 ± 2.1 kg/m^2^) participated in this single-blinded, two-arm randomized controlled trial (RCT). Participants were divided into WBS (*n* = 30) and control (*n* = 30) groups. The WBS group performed a range of stretching exercises targeting the entire body during a lunch break period for 3 times a week for 6 weeks. The control group received an education program. Musculoskeletal pain and physical exertion were assessed using the Nordic musculoskeletal questionnaire and Borg rating of perceived exertion scale, respectively. *Results*: The 12-month prevalence of musculoskeletal discomfort among all healthcare professionals was highest in the low back region (46.7%), followed by the neck (43.3%), and then the knee (28.3%). About 22% of participants said that their neck discomfort impacted their job, while about 18% reported that their low back pain impacted their job. Results indicate that the WBS and education program had a beneficial impact on pain and physical exertion (*p* < 0.001). When comparing the two groups, the WBS group experienced a significantly greater decrease in pain intensity (mean difference 3.6 vs. 2.5) and physical exertion (mean difference 5.6 vs. 4.0) compared to an education program only. *Conclusions*: This study suggests that doing WBS exercises during lunchtime can help lessen musculoskeletal pain and fatigue, making it easier to get through the workday.

## 1. Introduction

Work-related musculoskeletal disorders (WRMSDs) among healthcare professionals are prevalent and are costly public health problems across the globe [1,2,3]. WRMSDs typically manifest in the neck, lower back, and shoulder regions. According to the World Health Organization (WHO), WRMSDs might be aided by a person’s body type, working environment, and other psychosocial hazards [4]. Therefore, WRMSDs may be related to the conditions under which office workers perform their jobs. Particularly, healthcare professionals have a higher risk of developing musculoskeletal pain and injuries, especially related to the work they do in their daily routine [5,6,7,8]. The load and physical strain due to the manual handling of patients and prolonged awkward working postures in healthcare professionals can lead to many musculoskeletal symptoms, including pain and physical exertion [9,10]. Therefore, a preventive program is required to reduce musculoskeletal pain and physical exertion among healthcare professionals.

The major impact of WRMSDs is on the individual experiencing pain and discomfort, but there are also secondary effects on productivity due to the decreased quantity and quality of work accomplished by those who are impacted [11]. According to a recent review, physiotherapists, surgeons, and dentists are all at high risk for developing WRMSDs, with surgeons and dentists being more at risk [11]. They also showed that the lower back and neck are the areas most typically reported to be impacted by all three specialists. In addition, allied health professionals (AHPs), who play a crucial role in the health care system, had a high chance of having WRMSDs. Physical therapists (PTs), occupational therapists, speech pathologists, prosthetists, orthotists, dietitians, sonographers, social workers, osteopathic physicians, audiologists, radiologists, exercise physiologists, perfusionists, and, by some accounts, chiropractors, are all considered AHPs [12]. When compared to the general population, AHPs have a higher risk of developing WRMSDs because of the wide range of activities they perform at work and the hazards and risks to which they are exposed [13,14]. The duties of nurses and AHPs, for example, overlap significantly with those of other health care professionals. Both positions require a great deal of physical exertion and expose their holders to many psychological risks [13,14,15,16], such as heavy workloads, tight deadlines, or a lack of autonomy in their work. However, health care industry measures to reduce WRMSDs have generally concentrated on reducing the likelihood of physical hazards and risks, such as lifting or transporting patients [15,17]. Due in part to a lack of alignment between the cause variables of suspected WRMSDs and risk management techniques, the health care industry continues to report large numbers of WRMSDs despite significant attempts to limit their prevalence.

Lower back pain is more common among nurses because of their work with patients (transfers and repositioning), which involves frequent trunk flexion and rotation, awkward postures, and psychological and social stress [18]. Acute care PTs and PTs in rehabilitation settings face similar risks. Massage therapists have an elevated risk of developing digit-specific WMSDs [19]. Stress on the hands and thumbs is another risk for PTs who use manual techniques. One could assume that the prevalence of WMSDs among PTs would be lower than that of other professions undertaking patient handling responsibilities (such as nurses and massage therapists), given that PTs study injury prevention and are trained on body mechanics during their entry-level degree studies. The rate of WMSDs among PTs, however, has been shown to be similar to that of other health professionals involved in patient handling and transfers [19]. Within the first five years of practice, most PTs experience WMSDs [20,21]. While there is some individual research on WMSDs in PTs, only one systematic review of WMSDs in PTs was found. That review, however, appeared only in Polish [22].

Many treatment strategies have been described and studied in the literature to prevent WRMSDs [23,24,25]. Few studies, however, reveal long-term improvements in symptoms, claims, and disability outcomes. It has been proposed that healthcare workers would benefit from an exercise program at work to lessen the risk of WRMSDs and the associated pain and fatigue in muscles and joints [26,27,28]. Fragala et al. [29] conducted a review to provide evidence for the health advantages of resistance exercise. It was found that resistance training can slow or stop the onset of sarcopenia, muscle weakness, mobility loss, chronic disease, disability, and even early death. This publication includes guidelines for implementing resistance exercise regimens based on the best available evidence. Workplace treatment programs have been found to be effective in a variety of areas, including enhancing workers’ step counts, decreasing their inactivity time [30,31,32], and assisting workers in increasing their activity levels and losing weight [33]. Strength training in the workplace has been shown to minimize the incidence of WRMSDs among workers who do physically demanding jobs, according to a recent data synthesis [34]. Additionally, there was a deficiency of evidence in the scholarly literature to guide prevalent practices in workplace ergonomics [34]. To reduce WRMSDs among this workforce, it appears that participatory ergonomics and other comprehensive workplace interventions are ineffective [34]. Given the diversity of the areas in which interventions were implemented, it is important to proceed cautiously when drawing broad judgments regarding their efficacy [34]. More recently, Worley et al. [35] established the potential of hospital-based food and physical activity workplace interventions in influencing the health behavior of hospital workers. To better understand the advantages of workplace interventions in healthcare facilities, further research from high-quality, randomized control trials is needed [35]. Despite the magnitude of the problem posed by WRMSDs, studies and assessments have indicated that no single approach is particularly helpful [23,24,25].

Extensive prior research has established that stretching exercises reduce musculoskeletal discomfort [36,37,38]. As a result of the relaxing effects of stretching the affected muscles, the tension level in the affected area is indirectly reduced. Muscular pain caused by static stress can be alleviated with stretching [39,40]. By increasing the muscle pump, stretching can help boost blood flow to the affected muscles. As a result, spasm-related discomfort is alleviated, and the body’s ability to repair itself is enhanced [41,42,43]. Previous research has also shown that musculoskeletal pain risk can be reduced by engaging in regular physiotherapy training in the form of stretching with a frequency of 3–5 times a week [38]. Stretching during work has numerous positive effects. For instance, stretching helps maintain a healthy range of motion in the joints and a flexible, strong, and well-nourished muscle mass. Tightness and shortening of the muscles result from its absence. Then, when the time comes to put those muscles to work, they are too weak to fully expand. That can lead to issues including muscular strains, sprains, and joint pain. For instance, if individuals spend all day sitting, their hamstrings will become tight. Walking may be hampered because of the inability to fully extend the leg or straighten the knee. Similar damage can occur when previously tight muscles are suddenly asked to perform a strenuous stretching activity, such as participating in a sport. Damage to the joints can occur when the muscles supporting them are injured.

There have been numerous discussions about how to combat these problems by increasing physical activity and decreasing inactive time at work. Among the top priorities is getting office workers to become more physically active during their breaks [44]. Light-intensity physical exercises performed at the desk during regular breaks have been shown to improve workers’ health [45]. Healthcare professionals may benefit from less time spent sitting and more light physical activity throughout the day if daily schedules include activities such as short exercise interventions at the workplace [46]. The prevention and management of WRMSDs are possible outcomes of workplace exercise interventions [47]. Exercise interventions in the workplace can take several forms, from small breaks throughout the day to shortened exercises at the beginning and end of the workday and during the lunch break. Physical activity, such as stretching, is prevalent during brief breaks at the office and has been linked to enhanced mood and heightened muscle activity [48]. Stretching and strength exercise programs, either incorporating the whole body [49] or focused on a specific region such as the neck and shoulders [50], the trunk, or the lower limbs, are popular interventions in the workplace. The systematic review by Waongenngarm et al. [51] indicated that active breaks with posture modification reduced lower back pain and discomfort without negatively impacting the efficiency of the workers. In addition, a reduction in pain perception was seen in studies that looked at the effects of exercise interventions in the workplace for those experiencing symptoms of musculoskeletal illnesses [52,53,54]. The use of stretching to prevent work-related musculoskeletal problems and unintentional injuries is disputed, but a previous study showed some positive effects of stretching exercise programs in different occupations [55]. Therefore, the overarching aim of the proposed study is to investigate the effects of whole-body stretching (WBS) exercise during rest breaks on reducing musculoskeletal pain and physical exertion among healthcare professionals. This study hypothesizes that WBS exercise during rest breaks would significantly reduce musculoskeletal pain and physical exertion among healthcare professionals.

## 2. Materials and Methods

### 2.1. Study Design

**Figure 1:**
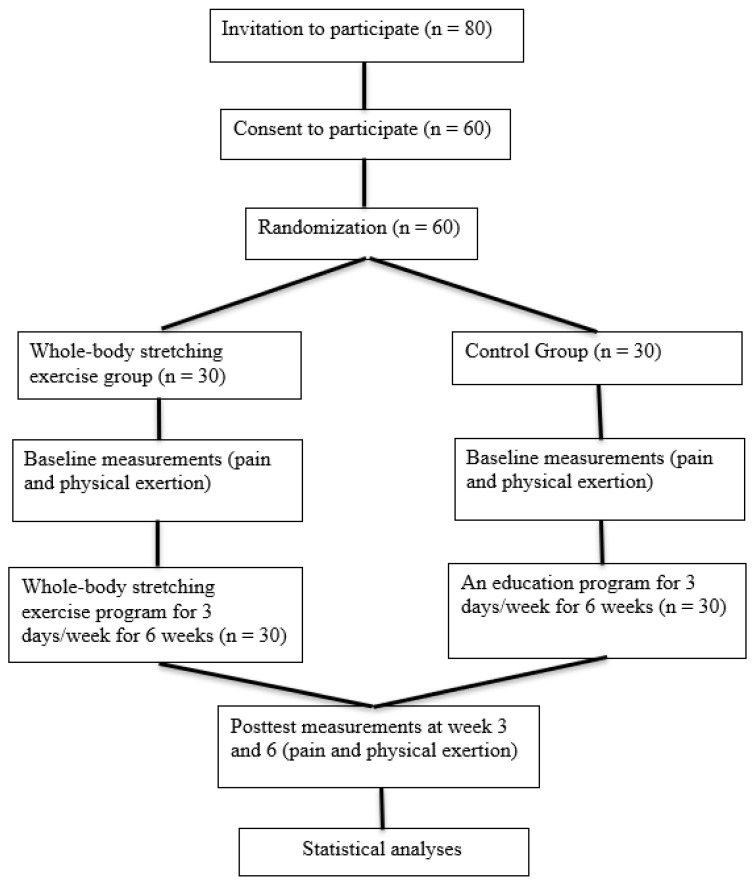
Flow diagram of the study procedures.

This study involved a single-blinded, 2-arm randomized controlled trial (RCT) (NCT05811715) to compare the effect of WBS exercise versus the control group during lunch breaks on reducing symptoms of WRMSDs in healthcare professionals (Figure 1). This trial has been designed as per the statement given by the Consolidated Standards of Reporting Trials [56].

### 2.2. Participants

Eligible healthcare professionals were recruited from Najran University Hospital, Saudi Arabia. Full-time healthcare professionals with more than one year of experience were eligible to participate. Individuals were excluded if they had any acute musculoskeletal symptoms (pain intensity on the visual analogue scale (VAS) > 7) that precluded participation in the exercise program. Participants were provided written informed consent approved by the institutional ethical review board at Najran University, Saudi Arabia (Reference No.: 444-37-25613-DS). Eligible participants were randomly allocated to either the WBS exercise group or the control group by an independent researcher using concealed random numbers in sealed, opaque envelopes [57]. Participants as well as assessors were blinded to the study allocation.

### 2.3. Interventions

For the WBS group, each 30-min exercise class was run by one trained physiotherapist to serve a maximum of 10 participants (10 × 3 = 30 participants) working in the hospital. Participants were invited to attend the exercise class three times a week for six weeks in their hospital during lunch breaks. During each session, participants were asked to perform WBS exercises (Table 1) [55,58]. The physiotherapists provided individualized exercise modification and progression and educated participants on the role of stretching to prevent musculoskeletal pain and physical exertion. Participants were encouraged to interact with one another during each session to strengthen their rapport and mutual support. Participants were instructed to report any increased pain or difficulty during the execution of the WBS exercise. Participants in the control group received an education program. An education program that included ergonomic principles, WRMSDs and their risk factors, as well as manual handling techniques, was explained [59].

### 2.4. Outcomes

Musculoskeletal pain and physical exertion were assessed using the Nordic musculoskeletal questionnaire (NMQ) [60,61,62] and the Borg rating of perceived exertion (RPE) [63,64], respectively. The NMQ is a standardized questionnaire for use in epidemiological studies that allows for the comparison of the lower back, neck, shoulder, and general symptoms [62]. There are three components to this questionnaire. In the first section, there is a generic questionnaire to identify the precise locations on the body where musculoskeletal issues manifest. With a body map, patients may pinpoint the location of their pain on their neck, shoulders, upper back, elbows, lower back, wrists, hands, hips, thighs, knees, and feet. In the second section, participants are questioned about any musculoskeletal issues they’ve experienced recently or over the past 12 months that have limited their daily activities. Finally, they are questioned about musculoskeletal injuries, accidents, functional impact at home and work, duration of the problem, and symptoms experienced in the past week. The Borg rating of perceived exertion (RPE) scale is an instrument that is utilized for the purpose of determining an individual’s level of effort and exertion, as well as their level of shortness of breath and fatigue while performing physical tasks [65]. A categorical matrix of numbered intervals (6–20) with equal distances between different perceptions of exertion, Borg’s scale is a useful tool for comparing individual responses to a given level of effort [66].

### 2.5. Statistical Analyses

The statistical analysis was carried out with the help of SPSS version 26.0 (SPSS Inc., Chicago, IL, USA). The descriptive data are displayed as a frequency distribution along with the mean and standard deviation for the items of the NMQ. The Shapiro–Wilk test was carried out to validate the normality of the score distribution. The impact of WBS and an education program on pain intensity and physical exertion at the end of week 6 was analyzed using a repeated measures analysis of variance (ANOVA). Additionally, we used a 2 × 3 repeated measures ANOVA with time (at baseline, week 3, and week 6 of the posttest), group (WBS and control), and the interaction effect (time × group), in addition to (time × gender), (time × occupation), and (time × gender × occupation), as our independent variables. The post hoc analysis was not performed if the main effect of the intervention was not significant. Alternatively, a post hoc analysis with the Bonferroni correction was performed on time if the main effect of time was statistically significant. The level of significance was chosen at *p* values less than 0.05.

To determine an adequate sample size, the software application G-Power 3.1 was utilized. A minimum sample size of 26 participants was required for each of the groups, with the alpha level, power, and effect size each being set at 0.05, 0.80, and 0.4, respectively. Considering an attrition rate of 15%, there were a total of 30 participants in each of the groups [67].

## 3. Results

**Figure 2:**
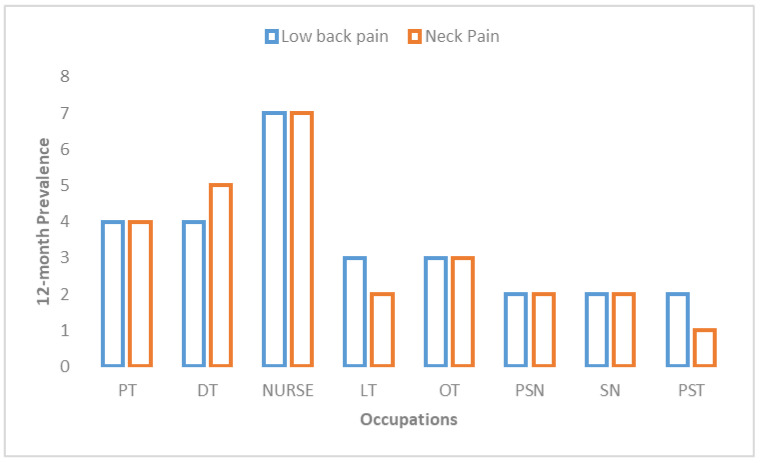
Twelve-month prevalence of low back pain and neck pain among healthcare professionals (Note: PT, physical therapists; DT, dental technicians; LT, laboratory technicians; OT, operation theater technicians; PSN, physician; SN, surgeon; PST, pharmacists).

**Figure 3:**
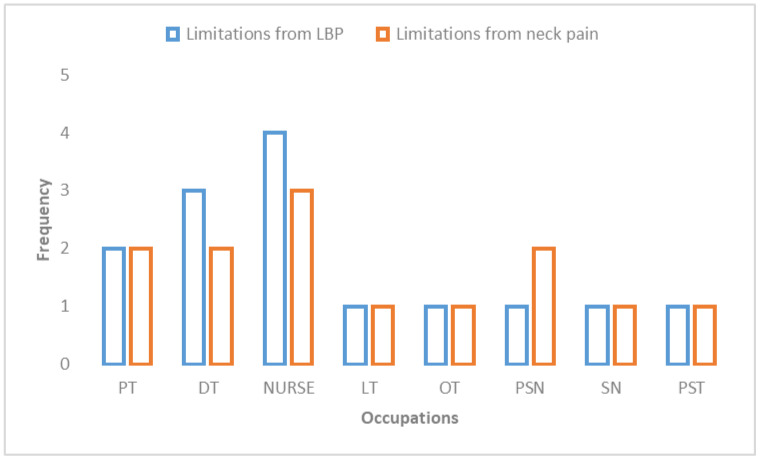
Limitations of work due to LBP and neck pain among healthcare professionals (Note: LBP, lower back pain; PT, physical therapists; DT, dental technicians; LT, laboratory technicians; OT, operation theater technicians; PSN, physician; SN, surgeon; PST, pharmacists).

Baseline characteristics (e.g., age, gender, height, body mass, etc.) of each group were compared. There was an insignificant difference noted between the two groups (*p* < 0.05). Table 2 details the descriptive data. Most participants belonged to nurses (25%), physiotherapists (20%), dental technicians (13.3%), and operation theater technicians (13.3%). Table 3 presents the prevalence of work-related musculoskeletal discomfort among healthcare professionals. The lower back region had the highest 12-month prevalence of self-reported musculoskeletal discomfort, with 46.7% of people experiencing it, followed by the neck, with 43.3% of people experiencing it, and then the knee, with 28.3% (Figure 2). Approximately 22% of participants reported that the discomfort in their neck impacted their job, and approximately 18% reported that the discomfort in their low back impacted their job (Table 4). According to the findings, both WBS and the education program had a positive effect on the patients’ level of pain and their level of physical exertion (*p* < 0.001) (Table 5 and Table 6). In a comparison of the two groups, the one that participated in WBS experienced a considerably higher reduction in pain intensity (mean difference 3.6 vs. 2.5) and physical exertion (mean difference 5.6 vs. 4.0) than the group that participated in the education program alone (Table 6). While there were no interaction effects of gender on outcome, the occupation of the participant impacted pain and physical exertion among healthcare professionals (Table 7; Figure 3).

## 4. Discussion

The purpose of this study was to examine the efficacy of lunchtime WBS exercise in reducing musculoskeletal pain and physical exertion among healthcare professionals. Nurses, physiotherapists, dental technicians, and operation theatre technicians were all shown to have significant rates of WRMSDs. The current study found that musculoskeletal pain was most common in the lower back, followed by the neck and the knee, over 12 months. Many of the study participants also mentioned that the pain in their lower backs and necks made it difficult for them to do their jobs. Similarly, earlier research has shown that the neck, shoulders, scapular area, shoulders, upper arms, and upper and lower backs are the most common sites of musculoskeletal symptoms among computer users [68,69,70]. These regions are also the ones that have the highest prevalence of affected individuals who work in call centers [71].

The lower back was the most common site of WRMSDs among healthcare professionals, acknowledging findings among PTs, nurses, and other health workers [72]. Around 47% of people had had lower back discomfort within the prior twelve months. When it comes to lower back pain, the prevalence among the 60 respondents was like that observed in other research (45% to 80%) [22,73], but greater than the prevalence seen in some other studies (26% to 29%) [74,75]. Possible explanations for the discrepancies include question wording and the timing of assessments (e.g., all-time versus during the last 12 months). Another study found that 68.1% of healthcare professionals had experienced musculoskeletal pain or discomfort in the previous year; this included 57.6% of doctors and 52.6% of nurses [76]. Additionally, nurses had the highest prevalence of lower back pain (77.1%), followed by doctors, physiotherapists, technicians, secretaries, and hospital aides, in a study examining the prevalence and causes of the condition among Turkish healthcare workers [77]. Like the current findings, 72 percent of the healthcare workers who reported musculoskeletal pain or discomfort said that it did not interfere with their work, while 28 percent reported that it did [76]. In addition, the results of the study are supported by the finding of a strong correlation between the occupation of healthcare workers and the WRMSDs [76,78].

Both WBS and the educational program for healthcare professionals resulted in a reduction in pain intensity and physical exertion. After 6 weeks of WBS exercise and the education program, pain intensity was reduced by 59.8% and 46.2%, respectively, compared to the baseline. After 6 weeks of WBS exercise and an education program, participants had a 34.9% and 26.1% decrease in physical exertion, respectively. A previous study also found a reduction in musculoskeletal discomfort and fatigue after a stretching program for call center operators [79]. In a similar vein, the frequency of pain was found to decrease after participants in other studies who assembled dental floss or worked as computer workers performed stretching and joint movement exercises [80,81,82]. Geneen et al. [83] found that physical activity reduced the onset and intensity of discomfort during prolonged activity. Nevertheless, they did not find that it had been eliminated altogether. However, Rasotto et al. [84] found no statistically significant differences in musculoskeletal complaints between those who did and did not take part in an exercise program. The training was carried out in the plant under the direction of a physical therapist as well as physical therapy assistants who acted as instructors and taught the various movements. It was not possible to evaluate compliance as well as the effectiveness or appropriateness of the exercises. Despite no reduction in pain, these authors found that 67% of individuals felt better after exercise, which is in line with our findings.

It is widely known that regular exercise helps improve physical health. For instance, Machado-Matos et al. [85] showed that emphasizing core stability when working out yields better results than just exercising in general. However, research by Robertson et al. [86] found that a simple modification to the height of the chair was more effective than strength training and stretching for relieving low back discomfort. Exercise training, however, can help office workers deal with their discomfort, leading to better sleep and less tiredness [87]. Active rest in the workplace has been shown to be beneficial to worker health and productivity by several different studies [88,89]. Consistent with past research, the results of this study support the argument. These results can be attributed to the potential benefits of implementing active rest inside the workplace during lunch breaks for the health and well-being of healthcare professionals.

WRMSDs have a detrimental effect on the productivity of healthcare workers, and they also contribute to a longer period of sick absence; therefore, the incidence of WRMSDs has wide-ranging repercussions for the economy of a country [90]. Due to the significant number of WRMSDs that occur in the workplace, it is imperative that the best possible preventative measures be identified. Healthcare professionals often face work-related risk factors such as bending, patient handling, performing repetitive tasks, and working in awkward body posture for prolonged periods of time. The prolonged working conditions of healthcare professionals are prone to musculoskeletal pain and physical exertion in these populations. A workplace WBS program could reduce the risk of developing various WRMSDs and minimize physical exertion in these populations. Stretching programs are helpful in reducing the incidence and/or severity of injuries by improving flexibility, motor control, and physical exertion. Healthcare professionals often report less flexibility and increased tiredness; therefore, they are more likely to have musculoskeletal pain and resultant injury. Therefore, the WBS program at the work site could be an effective program to improve flexibility and reduce musculoskeletal pain and physical exertion.

There are some limitations to this study. Results may not be generalizable because of the diverse sample of healthcare professionals. However, our findings provide useful information in this field of study and may influence future studies because this is one of the few investigations on these healthcare professionals available in the Arab and worldwide literature. Another possible shortcoming is that musculoskeletal complaints and physical exertion were measured using only subjective scales. However, such scales are widely used because the symptoms being measured (such as pain and exertion, which are subjective complaints) are so common and so well-established in the literature. Importantly, the researchers’ own biases did not influence the findings because the participants completed the surveys independently. Additionally, a few confounding factors, such as patient interaction and emotional factors, were not considered. Further investigations are required to corroborate our results on a larger scale and in a more homogenous group, considering these findings and the limitations of the current study. In addition to the subjective score employed here, future research should also incorporate objective measurements of physical performance and/or efficacy in the workplace.

## 5. Conclusions

This study found that healthcare workers who used their lunch breaks to do WBS exercises experienced less musculoskeletal pain and used less effort when doing a variety of physical tasks. However, more research is needed to confirm our findings on a larger scale and with a more consistent set of healthcare professionals, considering the current findings and limitations.

## Figures and Tables

**Table 1:** Details of stretching programs.

Sr. No.	Stretching Exercise Performed	Description
1	Neck stretch	Flexion, extension, and lateral flexion motions.
2	Shoulder stretch	Shoulder retraction, protraction, and elevation motions.
3	Arm curl	Biceps and Triceps stretch
4	Wrist bend	Wrist Flexor and extensor Stretch
5	Abdominal bend	Lying back extension
6	Trunk twist	Lumbar rotation
7	Hamstring stretch	Standing Hamstring stretch
8	Quad stretch	Standing Quadriceps stretch
9	Knee to chest	Back stretching
10	Side bends	Side stretching
11	Ankle circles	Ankle movement in circular manner
12	Ankle pumps	Plantar and dorsiflexor stretch

Note: Each stretch was held for 20 s with 5 repetitions.

**Table 2:** Descriptive data.

Variables	Frequency (%)
Age, mean (SD)	37.15 (3.9)
Gender,	
Male	34 (56.7)
Female	26 (43.3)
Height (m), mean (SD)	1.61 (0.04)
Body mass (kg), mean (SD)	67.8 (6.3)
Body mass index (kg/m^2^)	26.5 (2.1)
Occupation/Job	
PT	12 (20)
DT	8 (13.3)
Nurse	15 (25)
LT	6 (10)
OT	8 (13.3)
PSN	4 (6.7)
SN	4 (6.7)
PST	3 (5)
Working experience (months), mean (SD)	101.5 (39.3)
Working hours (per week), mean (SD)	57.4 (9.1)
VAS, mean (SD)	
Baseline	5.7 (1.05)
Week 3	4.1 (1.03)
Week 6	2.63 (1.06)
Borg RPE, mean (SD)	
Baseline	15.8 (1.6)
Week 3	13.5 (1.6)
Week 6	10.9 (1.7)

Note: VAS, visual analogue scale; RPE, rating of perceived exertion; PT, physical therapists; DT, dental technicians; LT, laboratory technicians; OT, operation theater technicians; PSN, physician; SN, surgeon; PST, pharmacists.

**Table 3:** Prevalence of work-related musculoskeletal disorders (WRMSDs) among healthcare professionals.

	12-Month Prevalence	1-Week Prevalence
Neck pain in last 12 months		
Yes	26 (43.3)	11 (18.3)
No	34 (56.7)	49 (81.7)
Shoulder pain in last 12 months		
Yes	15 (25)	7 (11.7)
No	45 (75)	53 (88.3)
Elbows pain in last 12 months		
Yes	10 (16.7)	4 (6.7)
No	50 (83.3)	56 (93.3)
Wrist/Hand pain in last 12 months		
Yes	14 (23.3)	6 (10)
No	46 (76.7)	54 (90)
Upper back pain in last 12 months		
Yes	16 (26.7)	5 (8.3)
No	44 (73.3)	55 (91.7)
Lower back pain in last 12 months		
Yes	28 (46.7)	8 (13.3)
No	32 (53.3)	52 (86.7)
One or both Hips/Thighs pain in last 12 months		
Yes	15 (25)	4 (6.7)
No	45 (75)	56 (93.3)
One or both Knees pain in last 12 months		
Yes	17 (28.3)	7 (11.7)
No	43 (71.7)	53 (88.3)
One or both Ankles/Feet pain in last 12 months		
Yes	13 (21.7)	3 (5)
No	47 (78.3)	57 (95)

**Table 4:** Impacts of work-related musculoskeletal pain among healthcare professionals.

	Frequency (%)
Neck pain prevented work in last 12 months	
Yes	13 (21.7)
No	47 (78.3)
Shoulder pain prevented work in last 12 months	
Yes	4 (6.7)
No	56 (93.3)
Elbows pain prevented work in last 12 months	
Yes	3 (5)
No	57 (95)
Wrist/Hand pain prevented work in last 12 months	
Yes	5 (8.3)
No	55 (91.7)
Upper back pain prevented work in last 12 months	
Yes	9 (15)
No	51 (85)
Lower back pain prevented work in last 12 months	
Yes	11 (18.3)
No	49 (81.7)
Hip/Thigh pain prevented work in last 12 months	
Yes	7 (11.7)
No	53 (88.3)
Knee pain prevented work in last 12 months	
Yes	5 (8.3)
No	55 (91.7)
Ankle/Feet pain prevented work in last 12 months	
Yes	2 (3.3)
No	58 (96.7)

**Table 5:** Effects of whole-body stretching (WBS) exercises and an education program (control group) on pain intensity (VAS scores) among healthcare professionals.

Groups	Baseline (A) Mean (SD)	Week 3 (B) Mean (SD)	Week 6 (C) Mean (SD)	ANOVA		Post hoc Analysis (Bonferroni)		
				F	* *p*	A vs. B	A vs. C	B vs. C
WBS	5.97 (1.21)	3.83 (1.15)	2.40 (1.16)	216.213	0.001 *	*p* < 0.05	*p* < 0.01 *	*p* < 0.05 *
Control	5.33 (0.76)	4.37 (0.76)	2.87 (0.90)	174.220	0.001 *	*p* > 0.05	*p* < 0.05 *	*p* < 0.05 *

Note: VAS, visual analogue scale; WBS, whole-body stretch; * Statistically significant *p* < 0.05.

**Table 6:** Effects of whole-body stretching (WBS) exercises and an education program (control group) on physical exertion (Borg RPE scores) among healthcare professionals.

Groups	Baseline (A) Mean (SD)	Week 3 (B) Mean (SD)	Week 6 (C) Mean (SD)	ANOVA		Post hoc Analysis (Bonferroni)		
				F	* *p*	A vs. B	A vs. C	B vs. C
WBS	16.13 (1.83)	13.17 (1.91)	10.50 (2.33)	161.140	0.001 *	*p* > 0.05	*p* < 0.01 *	*p* < 0.01 *
Control	15.40 (1.31)	13.80 (1.89)	11.40 (1.40)	286.488	0.001 *	*p* > 0.05	*p* < 0.05 *	*p* < 0.05 *

Note: RPE, rating of perceived exertion; WBS, whole-body stretch; * Statistically significant *p* < 0.05.

**Table 7:** Two-way (2 × 3) repeated measures analysis of variance.

Variables		Type III Sum of Squares	Mean Square	F	*p*
VAS	Time	232.63	116.32	352.96	0.001 *
	Time * Group	1.84	0.92	3.79	0.047 *
	Time * Gender	0.44	0.22	0.67	0.513
	Time * Occupation	9.13	0.65	1.98	0.029 *
	Time * Gender * Occupation	2.88	0.29	0.87	0.560
Borg RPE	Time	586.62	293.31	406.12	0.001 *
	Time * Group	6.52	3.26	4.51	0.014 *
	Time * Gender	0.77	0.38	0.530	0.590
	Time * Occupation	21.62	1.54	2.14	0.017 *
	Time * Gender * Occupation	5.64	0.56	0.780	0.647

Note: VAS, visual analogue scale; RPE, rating of perceived exertion; * Statistically significant *p* < 0.05.

## Data Availability

Data will be provided by the corresponding author.
